# PD‐L1 inhibitors combined with whole brain radiotherapy in patients with small cell lung cancer brain metastases: Real‐world evidence

**DOI:** 10.1002/cam4.7125

**Published:** 2024-04-12

**Authors:** Litang Huang, Shen Chen, Hui Liu, Lu Meng, Chengxing Liu, Xiaoting Wu, Yingying Wang, Shilan Luo, Hongbin Tu, Chunlei Wang, Ming Zhang, Xiaomei Gong

**Affiliations:** ^1^ Department of Radiation Oncology, Shanghai Pulmonary Hospital, School of Medicine Tongji University Shanghai China; ^2^ Department of Oncology, Shanghai Pulmonary Hospital Tongji University, School of Medicine Shanghai China; ^3^ Department of Cardiology, Tongji Hospital Tongji University, School of Medicine Shanghai China; ^4^ Department of Integrated TCM and Western Medicine, Shanghai Pulmonary Hospital Tongji University, School of Medicine Shanghai China; ^5^ Department of Endocrinology The Fourth Affiliated Hospital of Nantong University Jiangsu China; ^6^ Department of Integrated Traditional Chinese and Western Medicine Shanghai Jiao Tong University School of Medicine, Shanghai Chest Hospital Shanghai China

**Keywords:** extensive small cell lung cancer, immunotherapy, intracranial metastasis, radiotherapy

## Abstract

**Background:**

Numerous studies have demonstrated that brain metastases patients may benefit from intracranial radiotherapy combined with immune checkpoint inhibitors (ICIs). However, it is unclear whether this treatment is effective for patients with small cell lung cancer brain metastases (SCLC‐BMs).

**Methods:**

We conducted a retrospective study by analyzing medical records of patients with SCLC‐BMs from January 1, 2017 to June 1, 2022. Data related to median overall survival (mOS), median progression‐free survival (mPFS), and intracranial progression‐free survival (iPFS) were analyzed.

**Results:**

A total of 109 patients were enrolled, of which 60 received WBRT and 49 received WBRT‐ICI. Compared to the WBRT alone cohort, the WBRT‐ICI cohort showed longer mOS (20.4 months vs. 29.3 months, *p* = 0.021), mPFS (7.9 months vs. 15.1 months, *p* < 0.001), and iPFS (8.3 months vs. 16.5 months, *p* < 0.001). Furthermore, WBRT‐ICI cohort had a better response rate for both BMs. (*p* = 0.035) and extracranial diseases (*p* < 0.001) compared to those receiving WBRT alone. Notably, the use of WBRT before ICI was associated with longer mOS compared to the use of WBRT after ICI (23.3 months for the ICI‐WBRT group vs. 34.8 months for the WBRT‐ICI group, *p* = 0.020).

**Conclusion:**

Our results indicated that WBRT combined with immunotherapy improved survival in SCLC‐BMs patients compared to WBRT monotherapy. Administering WBRT prior to ICI treatment is associated with improved survival outcomes compared to WBRT following ICI treatment, for patients with SCLC‐BMs. These findings highlight the significance of conducting further prospective researches on combination strategies of intracranial radiotherapy and ICI in SCLC‐BMs patients.

## 1 INTRODUCTION

Small cell lung cancer (SCLC) is a malignant and aggressive tumor originating from the mucous membrane of the lungs or from the gland.^1^ Patients with SCLC often have poor clinical outcomes due to the high risk of the cancer spreading to the brain. Within 2 years of diagnosis, almost 80% of SCLC patients experience brain metastases (BMs).^2^ The median survival for patients with untreated SCLC‐BMs is approximately 3 months.^3^ Most BMs from SCLC are multifocal, making whole brain radiotherapy (WBRT) the preferred treatment option for patients with SCLC BMs.^4^ Previous researches have demonstrated that WBRT regimen improves the interval to central nervous system (CNS) progression in SCLC patients, with a median overall survival (mOS) of 5.2 months.^5^ Additionally, an analysis of the America National Cancer Database revealed that WBRT regimen was associated with a slight improvement in mOS for SCLC‐BMs patients age 75 or older.^6^ Recent clinical trials have shown that the combination therapy of PD‐L1 inhibitor and platinum‐based etoposide chemotherapy regimen is the preferred treatment for extensive stage SCLC. The IMpower133 and CASPIAN trials have also demonstrated that this therapy prolongs mOS to approximately 12 months and reduces the risk of death by 30% and disease progression (PD) by 23%.^7^, ^8^ Additional evidence revealed that patients benefited from the durvalumab regimen regardless of baseline BMs status, which suggested that PD‐L1 inhibitors contribute to improved survival in SCLC‐BMs patients.^8^

Based on preclinical findings, the brain transports cerebrospinal fluid containing immune cells via functional lymphatic vessels, and dendritic cells, T‐cells, and tumor‐associated macrophages invade the immune microenvironment of BM.^9^ Noticeably, immunomodulatory molecules, such as PD‐L1, have been detected within BMs originating from diverse tumor types.^10^, ^11^ A pooled analysis of the Keynote 158 and Keynote 028 trials revealed an objective response rate (ORR) of 15.4% among a subgroup of 13 patients with baseline BM who received immunotherapy. During the pembrolizumab maintenance after first‐line chemotherapy period, 22% of patients experienced BMs, with a mOS of 9.6 months, suggesting that ICIs were associated with survival benefits in SCLC‐BMs patients.^12^, ^13^

Radiotherapy reprograms the tumor immune microenvironment through multiple mechanism and synergizes with immunotherapy.^14^ For instance, radiation can enhance the antitumor effect of immunotherapy by activating dendritic cells, increasing the density of tumor‐infiltrating lymphocytes (TILs), and modulating PD‐L1 expression.^15^ In numerous clinical trials, radiotherapy in combination with immunotherapy has been shown to improve the clinical outcomes of cancer patients.^16^, ^17^, ^18^, ^19^ Results from a meta‐analysis also showed that radiotherapy combined with immunotherapy significantly outperformed radiotherapy alone in patients with lung cancer BMs.^20^ According to the results of a retrospective study, combined cranial radiotherapy and immunotherapy were correlated with improved survival benefits in patients with NSCLC, renal cell carcinoma (RCC) or melanoma intracranial metastases. The mOS was up to 18 months with cranial radiotherapy combined with immunotherapy.^21^ Briefly, the integration of radiotherapy and immunotherapy holds promising potential as a therapeutic approach, leading to improved survival outcomes in patients diagnosed with SCLC‐BMs. However, the specific role of WBRT‐ICI in SCLC‐BMs and whether the WBRT‐ICI regimen offers advantages over the WBRT alone in SCLC‐BMs is still unclear. Therefore, we conducted this study to assess the survival outcomes of WBRT‐ICI and WBRT alone in patients with SCLC‐BMs, aiming to provide optimized treatment strategies for SCLC‐BMs patients.

## 2 METHODS

### 2.1 Patients and eligibility criteria

A total of 109 patients with SCLC‐BMs at two centers between January 1, 2017 and June 1, 2022 were enrolled. Among them, the patients of WBRT cohort were all from Shanghai Pulmonary Hospital, 10 patients in the WBRT‐ICIs cohort were from Shanghai Chest Hospital, and the rest were from Shanghai Pulmonary Hospital. Inclusion criteria were: (1) pathologically confirmed small cell lung cancer; (2) the presence of BMs on cranial MRI; (3) sufficient information about follow‐up and electronic medical records; (4) adopted WBRT‐ICIs or WBRT treatment; (5) ≥18 years old; (6) received more than 3 cycles of immunotherapy; (7) extracranial lesions were stably controlled. Exclusion criteria were: (1) combined with other tumors; (2) received prophylactic cranial irradiation (PCI) or any type of surgery; (3) follow‐up not completed; (4) death without imaging evidence of disease progression. The before group included sequential therapy (WBRT within 3 months prior to initial immunotherapy), and the after group include induction therapy (several rounds of immunotherapy followed by concurrent WBRT) and concurrent therapy (concurrent WBRT and immunotherapy).

The time period from the initial diagnosis of BMs until the date of death or the last follow‐up, which occurred on January 30, 2023, was referred to as overall survival (OS). Progression‐free survival was calculated from the start of immunotherapy to the date of progression disease (According to Response Evaluation Criteria in Solid Tumors (RECIST) version 1.1) or was censored at the date of last CT when no progression had occurred. Available follow‐up MRI imaging and reports were reviewed for new BMs, disease progression, leptomeningeal disease at most every 3 months per institutional practice until death, hospice enrollment, or leaving the hospital refusing any resuscitation. Intracranial progression‐free survival (iPFS) was defined as the interval between WBRT and their earliest CNS progression or as the date at which they went on to have last MRI if there was no CNS progression (According to RANO‐BM criteria: assessment of response to neurotumor BMs). RANO‐BM criteria defined as pathologically confirmed or a clinician's decision to change treatment due to imaging progression of previously treated BMs. Follow‐up MRIs were obtained at 1, 3, 6, 9, and 12 months and lesion volumes were calculated at all available time points. To determine intracranial disease response, the maximum diameter of the T1 enhanced portion of all lesions was measured in three orthogonal planes at the time of treatment and at each follow‐up and the lesion volume was calculated as (length × width × height)/2. RANO‐BM criteria were used to define complete response (CR), partial response (PR), disease stabilization (SD), and PD.

**FIGURE 1:**
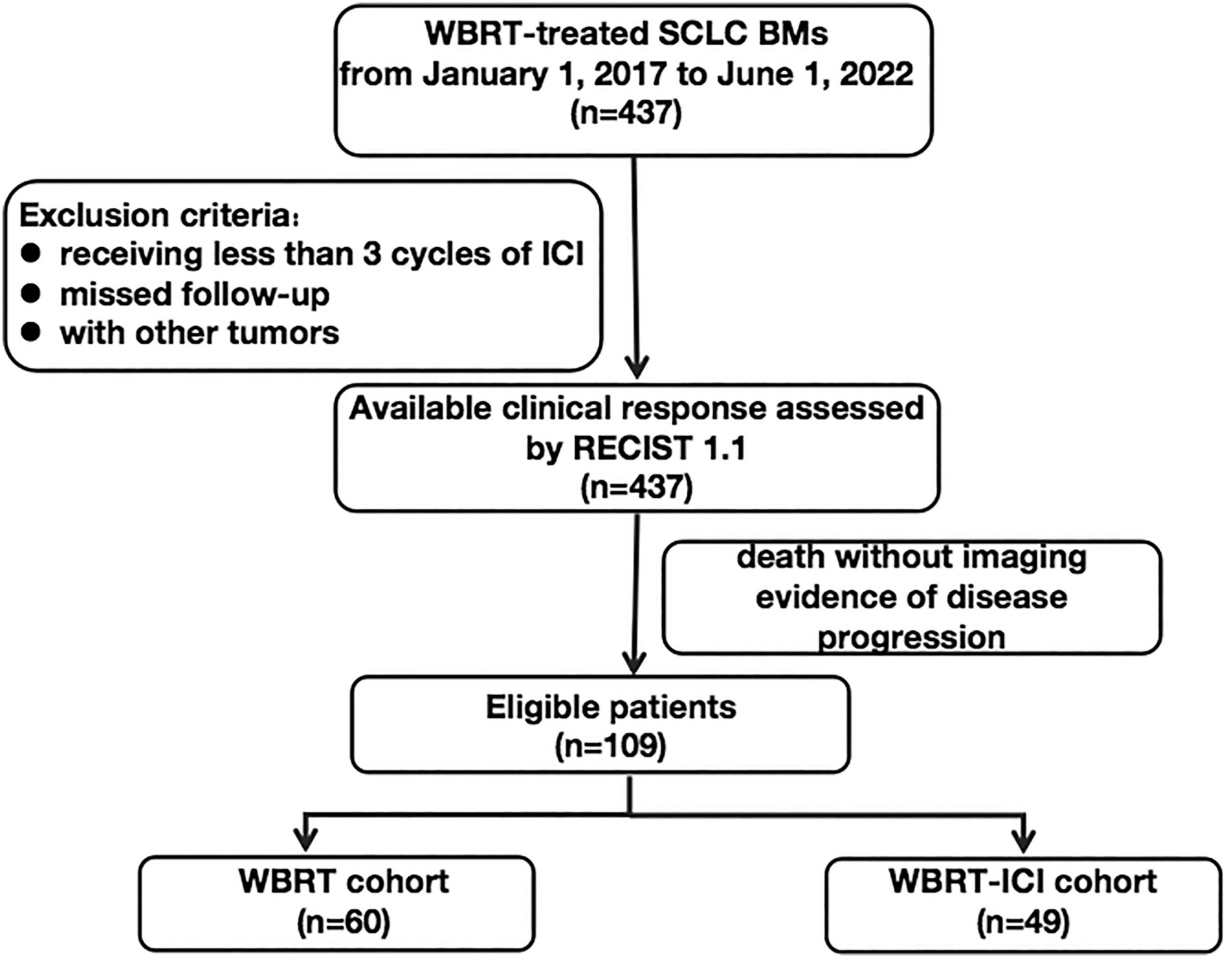
Flow chart of patients recruiting.

Our study was approved by the institutional review boards of the two participating centers, which granted an informed consent waiver. The protocol was approved by the Ethics Review Committee of Shanghai Pulmonary Hospital. The approval number is L22‐304. The study followed the Declaration of Helsinki (as revised in 2013) and deidentified and anonymized all patient records prior to analysis. Figure 1 depicts the recruitment flow chart for the cohorts of patients.

### 2.2 Treatment regimen

All patients underwent WBRT. WBRT was performed with 3 Gy/time for 10 times, and the total dose in 2 weeks was 30 Gy. For the immunotherapy strategy, patients treated with PD‐L1 inhibitors (atezolizumab: initially 1200 mg every 21 days × 4 cycles, then 1200 mg every 21 days for maintenance; durvalumab: initially 1500 mg every 21 days × 4 cycles, then 1500 mg every 21 days for maintenance).^4^

### 2.3 Statistical analysis

To compare clinical parameters between WBRT and WBRT‐ICIs cohort, we used Fisher's exact test or chi‐squared test. An estimate of survival (mOS, mPFS, and iPFS) was determined by Kaplan–Meier, and comparisons were made using log‐rank tests. To assess the relationship with mOS, we used Cox proportional hazards regression.

The association between factors and mOS were evaluated by Cox proportional hazards regression. SPSS 23.0 was used for all analyses. The significance level was set at *p* ≤ 0.05, and all *p*‐values were based on a two‐sided significance level.

## 3 RESULTS

### 3.1 Descriptions of the patients' characteristics

The ultimate analysis included 109 patients with SCLC‐BMs, including 60 patients in the WBRT cohort and 49 patients in the WBRT‐ICI cohort. Table 1 presents the demographics and clinical characteristics of two groups. Patients were on average 61.2 ± 8.9 years old (range: 39–85 years old); 41.3% (45/109) were ≥ 65 years old. Only 6.4% (7/109) of the patients were female. A total of 101 patients (92.7%) had an ECOG‐PS 0–1 and only eight (7.3%) had an ECOG‐PS ≥2. The proportion of BMs greater than three was similar between the two cohorts. Most patients (88/109) had BMs with a maximum diameter of more than 1 cm. The proportion of Karnofsky performance status (KPS) score higher than 80 was 81.7% in WBRT group and 77.6% in WBRT‐ICI group. The majority of patients had a Graded Prognostic Assessment (GPA) score of 0–2 (86/109), whereas 21.1% patients (23/109) were greater than 2. The patients of WBRT treated cohort were all exposed to platinum‐based doublet chemotherapy. The mentioned characteristics were well equated between two groups.

**TABLE 1:** Baseline demographic and clinical characteristics of patients with SCLC brain metastasis.

	Total (*N* = 109)	WBRT cohort (*N* = 60)	WBRT‐ICI cohort (*N* = 49)	*p*
Age, years				
<65	64 (58.7%)	35 (58.3%)	29 (59.2%)	1.000
≥65	45 (41.3%)	25 (41.7%)	20 (40.8%)	
Sex				
Female	7 (6.4%)	4 (6.7%)	3 (6.1%)	1.000
Male	102 (93.6)	56 (93.3%)	46 (93.9%)	
Smoking				
Yes	63 (57.8%)	34 (56.7%)	29 (59.2%)	0.944
No	46 (42.2%)	26 (43.3%)	20 (40.8)	
ECOG‐PS				
0–1	101 (92.7%)	54 (93.3%)	45 (91.8%)	1.000
≥2	8 (7.3%)	4 (6.7%)	4 (8.2%)	
No. of BMs				
≤3	54 (49.5%)	35 (58.3%)	19 (38.8%)	0.66
>3	55 (50.5%)	25 (41.7%)	30 (61.2%)	
Max tumor diameter in cm				
<1	21 (19.3%)	11 (18.3%)	10 (20.4%)	0.977
≥1	88 (80.7%)	49 (81.7%)	39 (79.6%)	
Extracranial metastases				
Yes	59 (54.1%)	29 (48.3%)	30 (61.2%)	0.246
Bone		12	17	
Adrenal gland		13	5	
Lymphoglandula		6	6	
Liver		5	11	
No	50 (45.9%)	31 (51.7%)	19 (38.8%)	
KPS score				
	22 (20.2%)	11 (18.3%)	11 (22.4)	0.770
≥80	87 (79.8%)	49 (81.7%)	38 (77.6%)	
GPA score				
≤2	86 (78.9%)	43 (71.7%)	43 (87.8%)	0.070
>2	23 (21.1%)	17 (28.3%)	6 (12.2%)	
RPA score at WBRT				
Class I	16 (14.7%)	5 (8.3%)	11 (22.4%)	0.113
Class II	90 (82.6%)	53 (88.3%)	37 (75.5%)	
Class III	3 (2.8%)	2 (3.3%)	1 (2.0%)	
Platinum‐based doublet chemotherapy				
Yes	1 (0.9%)	0 (0.0%)	1 (2.0%)	0.919
No	108 (99.1%)	60 (100.0%)	48 (98.0%)	

Abbreviations: ECOG‐PS, Eastern Cooperative Oncology Group performance status; GPA, graded prognostic assessment; KPS, Karnofsky performance status; RPA, recursive partition analysis; SCLC‐BM, small cell lung cancer brain metastasis; WBRT, whole brain radiotherapy; WBRT‐ICI, whole brain radiotherapy‐immune checkpoint inhibitors.

### 3.2 Survival analyses

**FIGURE 2:**
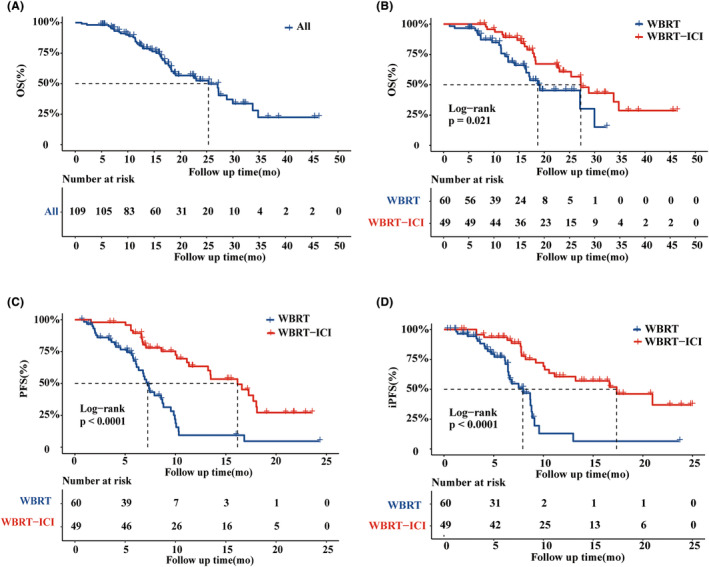
(A) Kaplan–Meier curve for median overall survival (mOS) for all patients, (B–D) median OS, progression‐free survival (PFS) and intracranial progression‐free survival (iPFS) in the WBRT and WBRT‐ICI group.

Median OS from all included patients was 26.2 months (95% CI, 22.6–29.7). (Figure 2A) For the WBRT‐ICI group, the median follow‐up time was 17.2 months (95% CI, 14.7–20.5 months) while for the WBRT group, the median follow‐up time was 26.9 months (95% CI, 22.5–31.7 months). Patients treated with WBRT‐ICI had a mOS of 29.3 months (95% CI, 24.8–33.9 months), and those treated with WBRT had a mOS of 20.4 months (95% CI, 17.3–23.4 months). The difference in the mOS between the two groups was significant (log‐rank *p* = 0.021) (Figure 2B). There was a significant improvement in median PFS in the WBRT‐ICI group (15.1 months, 95% CI, 12.9–17.2 months) as compared to the WBRT group (7.9 months, 95% CI, 6.3–9.4 months; stratified HR 3.6, 95% CI 2.1–6.1; log‐rank *p* < 0.001) (Figure 2C). We performed iPFS analyses in 109 patients with clinical follow‐up data to assess CNS control. In all patients, at least one follow‐up brain MRI examination was performed after the WBRT. Accordingly, in the WBRT‐ICI cohort, the median iPFS was 15.1 months (95% CI, 12.9–17.2 months) compared to 7.9 months (95% CI, 6.3–9.4 months) for the WBRT cohort (log‐rank *p* < 0.001) (Figure 2D).

**FIGURE 3:**
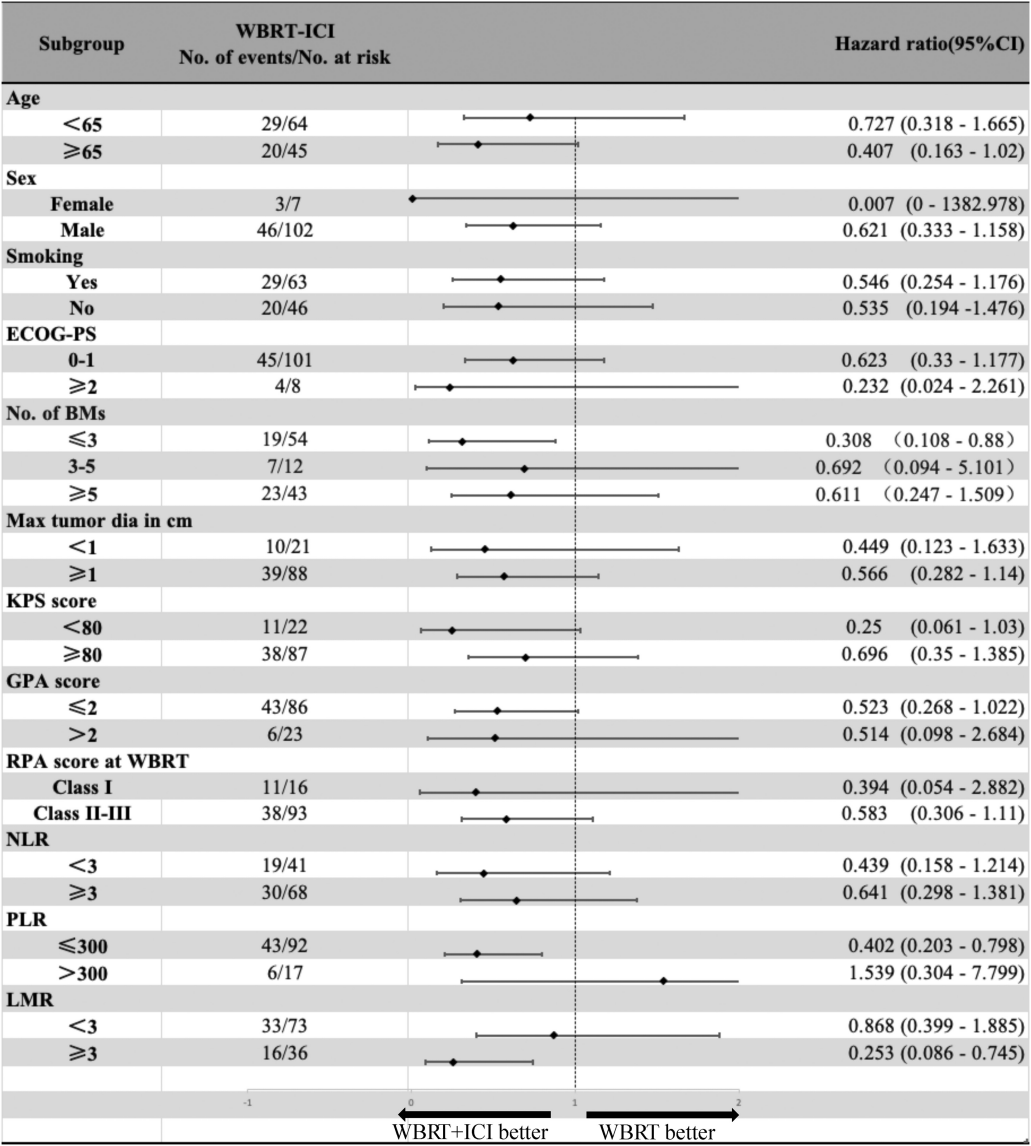
Subgroup analysis of overall survival (OS). All patients diagnosed with small cell lung cancer brain metastases (SCLC‐BMs). ECOG‐PS, Eastern Cooperative Oncology Group performance status; GPA, Graded prognostic assessment; KPS, Karnofsky performance status; LMR, lymphocyto‐to‐monocyto ratio; NLR, neutrophil‐to‐lymphocyte ratio; PLR, platelet‐to‐lymphocytopy ratio; RPA, recursive partition analysis.

Exploratory subgroup analysis found that effect size directions were consistent across age, sex, smoking status, ECOG‐PS, numbers of BMs, max tumor diameter in centimeter, KPS score, GPA score, RPA score at WBRT, baseline neutrophil‐to‐lymphocyte ratio (NLR), platelet lymphoid ratio (PLR) as well as lymphocyte‐to‐monocyte ratio (LMR) subgroups (Figure 3). Patients with three BMs or less benefited from WBRT‐ICI treatment with a prolonged OS (HR = 0.3, 95% CI 0.108–0.88) but there was no significant correlation between WBRT‐ICI regimen and mOS in patients with more than three BMs lesions (HR = 0.724, 95% CI, 0.323–1.619).

The group with a lower platelet‐to‐lymphocyte ratio (PLR ≤300) could also benefit from the WBRT‐ICI regimen (HR = 0.402, 95% CI 0.203–0.798). However, the group with a higher PLR (PLR >300) could not (HR = 1.539, 95% CI 0.304–7.799). Higher lymphocyte‐to‐monocyte ratio (LMR ≥3) group also experienced prolonged OS (HR = 0.253 95% CI 0.086–0.745) but not the lower LMR group (HR = 0.868 95% CI, 0.399–1.885) (Figure 3).

### 3.3 Tumor response

**FIGURE 4:**
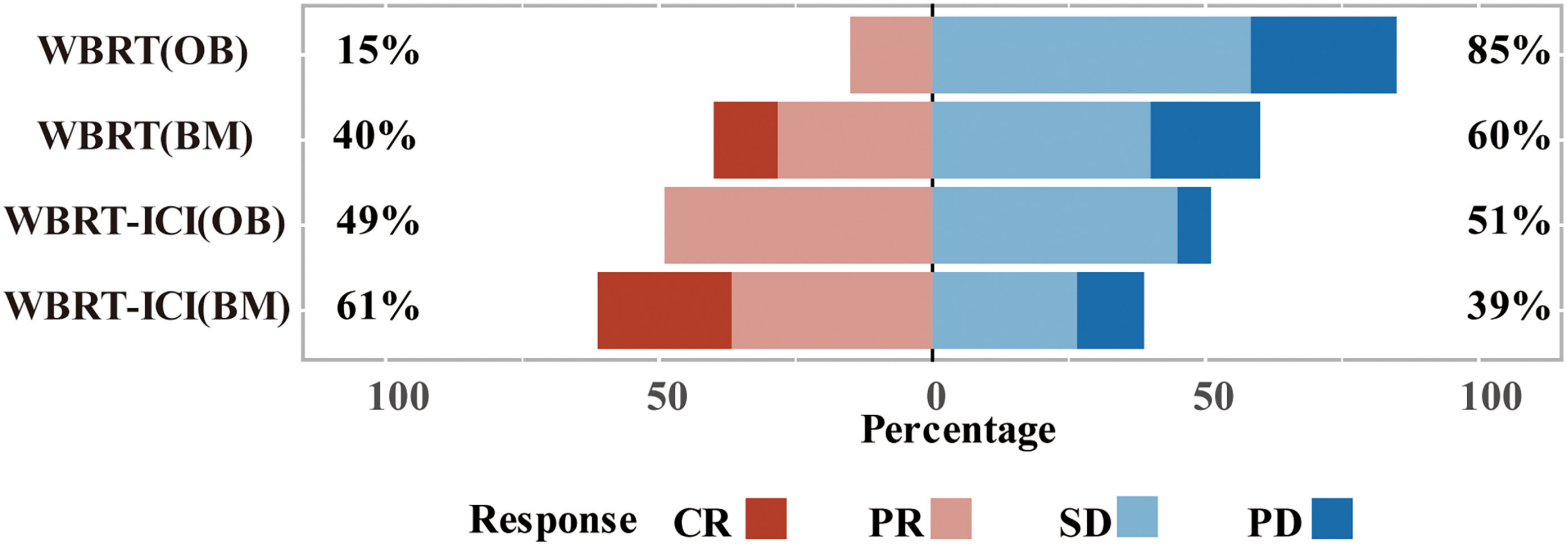
Best objective tumor response of patients evaluated by RECIST 1.1 criteria. Bars indicated best response for patients. BM, brain metastases; CR, complete response; OB, outside of brain; PD, progressive disease; PR, partial response; SD, stable disease.

The best responses to treatment of extracerebral disease and the BMs of the included patients were presented in Figure 4. The proportion of CR in the brain was 24.5% in the WBRT‐ICI cohort, which was higher than that seen in the WBRT cohort (11.6%). Moreover, higher progressive disease (PD) rate was observed in patients with WBRT (20%), compared to patients with WBRT‐ICI (12.2%).

The tumor response of anti‐tumor therapy was stratified as response group (CR and PR) and no response group (stable disease [SD] and PD). There was a significant difference of intracranial response rate between WBRT cohort and WBRT‐ICI cohort (*p* = 0.035). Similarly, we found that the response rate of extracerebral disease in the WBRT‐ICI cohort is statistically higher than that in WBRT cohort (*p* < 0.001; Table 2).

**TABLE 2:** Clinical effectiveness of patients. Response contained duration of complete response (CR) and partial response (PR); no response contained duration of stable disease (SD) and progressive disease (PD).

	WBRT (*N* = 60)	WBRT‐ICI (*N* = 49)	*p*
	No. of patients (%)	No. of patients (%)	
Best response of brain metastases			
Response	24 (40%)	30 (61%)	0.035
No response	36 (60%)	19 (39%)	
Best response of outside the brain			
Response	9 (15%)	24 (49%)	0.000
No response	51 (85%)	25 (51%)	

### 3.4 Timing of radiotherapy

**FIGURE 5:**
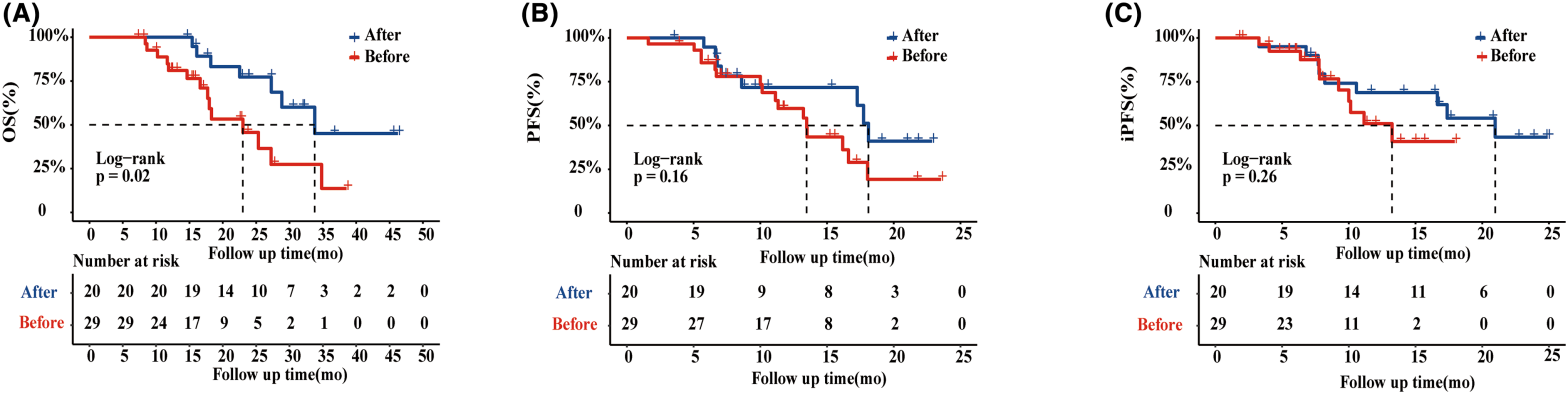
(A) Kaplan–Meier curve for overall survival (OS), (B) progression‐free survival and (C) intracranial progression‐free survival (iPFS), stratified by timing of WBRT.

The enrolled patients were split into two groups based on the time window in which the WBRT was administered. There were 29 patients in the before group and 20 patients in the after group. A comparison of the clinical characteristics of the two groups can be seen in Table 3. Notably, a significant difference in median OS was seen between the before and after group (34.8 months vs. 23.3 months, log‐rank *p* = 0.020; Figure 5A). According to these results, a delayed WBRT treatment was associated with a worse OS than an earlier WBRT treatment. The WBRT‐ICI (before group) regimen demonstrated a non‐significant superior efficacy on PFS (log‐rank *p* = 0.167) and iPFS (log‐rank *p* = 0.263) compared to the after group (Figure 5B,C ).

**TABLE 3:** Baseline characteristics in WBRT‐ICI population, stratified by timing of WBRT.

	Total (*N* = 49)	After group (*N* = 20)	Before group (*N* = 29)	*p*
Age				
<65	29 (59.2%)	13 (65.0%)	16 (55.2%)	0.695
≥65	20 (40.8%)	7 (35.0%)	13 (44.8%)	
Sex				
Female	3 (6.1%)	1 (5.0%)	2 (6.0%)	1.000
Male	46 (93.9)	19 (95.0%)	27 (93.1%)	
Smoking				
Yes	29 (59.2%)	15 (75.0%)	14 (48.3%)	0.115
No	20 (40.8%)	5 (25.0%)	15 (51.7%)	
ECOG‐PS				
0–1	45 (91.8%)	19 (95.0%)	26 (89.7%)	0.888
≥2	4 (8.2%)	1 (5.0%)	3 (10.3%)	
No. of BMs				
≤3	19 (38.8%)	8 (40.0%)	11 (37.9%)	1.000
>3	30 (61.2%)	12 (60.0%)	18 (62.1%)	
Max tumor diameter in cm				
<1	10 (20.4%)	3 (15.0%)	7 (24.1%)	0.675
≥1	39 (79.6%)	17 (85.0%)	22 (75.9%)	
KPS score				
<80	11 (22.4%)	3 (15.0%)	8 (27.6%)	0.491
≥80	38 (77.6%)	17 (85.0%)	21 (72.4%)	
GPA score				
≤2	43 (87.8%)	19 (95.0%)	24 (82.8%)	0.400
>2	6 (12.2%)	1 (5.0%)	5 (17.2%)	
RPA score at WBRT				
Class I	11 (22.4%)	4 (20.0%)	7 (24.1%)	0.647
Class II	37 (75.5%)	16 (80.0%)	21 (72.4%)	
Class III	1 (2.0%)	0 (0.0%)	1 (3.4%)	
Platinum‐based doublet chemotherapy				
Yes	48 (98.0%)	20 (100.0%)	28 (96.6%)	1.000
No	1 (2.0%)	0 (0.0%)	1 (3.4%)	

## 4 DISCUSSION

WBRT combined with PD‐L1 inhibitors for SCLC patients with BMs is promising and attractive. Retrospective data has shown that combining WBRT with ICI is significantly associated with better survival and tumor response in patients with SCLC‐BMs. Furthermore, an earlier regimen of WBRT was found to be superior in terms of OS compared to a delayed group. However, there is limited available data on the efficacy of combining intracranial radiotherapy and PD‐L1 inhibitors in SCLC‐BMs patients. Several prospective trials are currently underway or planned to evaluate the safety and efficacy of intracranial radiotherapy and ICI in treating BMs (NCT04787185, NCT03807765, NCT04889066, and NCT02662725).^22^, ^23^, ^24^, ^25^, ^26^ To our knowledge, there is a randomized controlled trial investigating the efficacy of combining radiosurgery and nivolumab in BMs among various tumors, including SCLC, although no published data is available (NCT02978404). Notably, several retrospective studies have observed that BMs patients could benefit from the combination treatment of intracranial radiotherapy and ICIs.^27^, ^28^, ^29^ For example, researchers have published a study of 199 melanoma and NSCLC patients who received concurrent or non‐concurrent ICI and radiotherapy, and noted that concurrent ICI and radiotherapy was associated with a lower proportion of PD and local recurrence.^19^ Claire M. reported a better OS and lower 1‐year cumulative incidence of neurologic death among 101 lung cancer and melanoma patients who received immunotherapy and stereotactic radiotherapy (SRS), compared to patients treated with SRS alone.^30^ Therefore, we conducted this study to explore the feasibility of combination of ICI and WBRT for SCLC‐BMs patients.

Inflammation plays a key role in the occurrence and development of tumors and is an important factor in assessing the prognosis of tumor patients.^31^, ^32^, ^33^ Several novel serum inflammatory markers, including NLR, PLR, LMR, and C‐reactive protein (CRP), have been shown to predict the risk of tumor recurrence and metastasis, and to assess the survival probability of patients with advanced lung cancer.^34^, ^35^, ^36^, ^37^ The predictive value of PLR and LMR in efficacy of immunotherapy has been reported in NSCLC, nasopharynx cancer, melanoma, RCC patients receiving immunotherapy or radiotherapy,^38^, ^39^, ^40^ and could be an independent prognostic factor among patients receiving ICI. The prognostic value of LMR has also been demonstrated in patients with SCLC. In our cohort, patients with low PLR (≤300) and high LMR (≥3) seemed to have superior OS when treated with a combination of intracranial radiotherapy and ICI. These findings imply that PLR and LMR may be potential indicators of the effectiveness of combination therapy in SCLC‐BMs patients receiving ICI plus intracranial irradiation.

Our results showed that the efficacy of WBRT‐ICI on SCLC‐BMs patients appears to be better when WBRT is performed before ICI (23.6 months for the before group vs. 12.0 months for the after group, log rank *p* = 0.04). Similar results were observed in previous studies. Results from 46 patients with melanoma‐BMs who received SRS‐Ipilimumab indicated that receiving intracranial radiotherapy before ICI is significantly related to lower 1‐year regional recurrence (69% for during ICI vs. 64% for before ICI vs. 92% for after ICI, *p* = 0.003) and longer 1‐year OS (65% for during ICI vs. 56% for before ICI vs. 40% for after ICI, *p* = 0.008).^41^ Moreover, Cohen‐Inbar et al. demonstrated that the 1‐year survival rate for intracranial radiotherapy administered during or prior to ICI and intracranial radiotherapy administered after ICI were 59% and 33%, respectively. Notably, the local recurrence‐free duration was higher in the SRS during and before cohort than in the SRS after group as well (19.6 months in during and before ipilimumab cycles and 3 months in after ipilimumab cycles, *p* = 0.005).^42^ Additionally, according to the timing of intracranial radiotherapy, a meta‐analysis divided 218 patients with BMs from different studies into three groups—ICI before SRS, ICI concurrent with SRS, and ICI after SRS. Pooled results demonstrated that ICI before SRS was associated with inferior 1‐year OS.^43^ Mechanistically, the release of antigens from dying tumor cells after intracranial radiotherapy may offer a synergistic antitumor effect with ICI.^14^ It is worth noting that “abscopal effect” of radiotherapy rarely occurred before the use of ICI. After the application of immunotherapy, higher rates of “abscopal effect” can be observed. As a result, we hypothesized that performing intracranial radiotherapy before immunotherapy can generate neoantigens that yield an “abscopal effect” which enhanced the anti‐tumor efficacy.^44^

A multicenter retrospective study suggested that compared to WBRT, SRS improved OS (8.5 months for SRS vs. 5.2 months for WBRT, log‐rank *p*<0.001).^45^ Clinical trials are ongoing to evaluate the efficacy of SRS and WBRT among SCLC‐BMs patients (NCT03297788, NCT03391362). There is still more work to be done in figuring out the best treatment plan for SCLC‐BMs patients, particularly for those who have brain oligometastases. Though it is the first studies examining the combination of WBRT and ICI for SCLC‐BMs patients, the study's validity may be constrained by its retrospective nature and small sample size. The quality of the medical records and clinical assessments was limited by the retrospective design, so data on treatment‐related adverse effects were insufficient. Therefore, prospective studies are urgently needed to further investigate the efficacy and safety of the combination of intracranial radiotherapy and ICI among the SCLC‐BMs patients.

Our study was a retrospective analysis based on real‐world data, in which we observed that a subset of patients with longer survival contributed to the prolonged median survival time in our cohort. This phenomenon may reflect the heterogeneity and complexity of the disease, as well as the individualized response to the treatment. However, our study had several limitations, such as the small sample size, the retrospective design, and the potential selection bias. Therefore, our findings need to be confirmed and validated in larger and prospective cohorts with more rigorous methodology and more representative population.

## 5 CONCLUSIONS

In conclusion, WBRT combined with PD‐L1 inhibitors exhibited a superior survival outcome in SCLC‐BMs patients compared to WBRT alone. Of note, the administration of WBRT prior to ICI is associated with improved survival outcomes compared to WBRT following ICI treatment in patients with SCLC‐BMs. More prospective studies are desired to verify the efficacy intracranial radiotherapy and ICI combining strategy in SCLC‐BMs patients.

## AUTHOR CONTRIBUTIONS

**Litang Huang:** Conceptualization (equal); data curation (lead); formal analysis (lead); writing‐original draft (lead). **Shen Chen:** Data curation (equal); formal analysis (equal); writing‐review and editing (equal). **Hui Liu:** Data curation (equal); methodology (equal); software (equal). **Lu Meng:** Data curation (equal); writing‐review and editing (equal). **Chengxing Liu:** Data curation (equal); writing‐review and editing. **Xiaoting Wu:** Formal analysis (equal). **Yingying Wang:** Data curation (equal). Shilan Luo: Investigation (equal). **Hongbin Tu:** Formal analysis (equal). **Ming Zhang:** Data curation (equal). **Xiaomei Gong:** Conceptualization (lead); data curation (equal); writing‐review and editing (equal); funding acquisition (equal); project administration (equal). **Chunlei Wang:** writing‐review and editing (equal).

## FUNDING INFORMATION

This work was supported by Shanghai Municipal Health Commission (202140256), Shanghai Science and Technology Innovation Action Plan (20Y11913600), Shanghai Natural Science Foundation (21ZR1453300), Shanghai Pulmonary Hospital Backbone Program (fkgg1808), Shanghai Talents Development Fund Project (2021071), Clinical Research foundation of Shanghai Pulmonary Hospital (SKPY2021006).

## CONFLICT OF INTEREST STATEMENT

The authors have no relevant financial or non‐financial interests to disclose.

## CONSENT

Informed consent was obtained from all individual participants included in the study.

## Data Availability

The raw data supporting the conclusions of this manuscript will be made available by the authors, without undue reservation, to any qualified researcher. Requests to access the datasets should be directed to Dr. Xiaomei Gong. Email: gongxiaomei1981@163.com.
